# Reviewer acknowledgement 2015

**DOI:** 10.1186/s12969-016-0065-9

**Published:** 2016-02-01

**Authors:** Alberto Martini, Charles Spencer

**Affiliations:** University of Genoa, Genoa, Italy; Ohio State University, Columbus, USA

## Abstract

The Editors of *Pediatric Rheumatology* would like to thank all our reviewers who have contributed to the journal in Volume 13 (2015).

**Rabheh Abdul Aziz**

United States of America

**Alexa Adams**

United Kingdom

**Amita Aggarwal**

India

**Haluk Akin**

Turkey

**Daniel Albert**

United States of America

**Jordi Anton**

Spain

**Monica Ardura**

United States of America

**Wineke Armbrust**

Netherlands

**Tadej Avcin**

Slovenia

**Sevcan Bakkaloglu**

Turkey

**Michael Beresford**

United Kingdom

**Peter Berlit**

Germany

**Peter Blier**

United States of America

**John Bohnsack**

United States of America

**Riva Brik**

Israel

**Paul Brogan**

United Kingdom

**Juergen Brunner**

Austria

**Ruben Burgos-Vargas**

Mexico

**Jon Burnham**

United States of America

**Daniela Cabibi**

Italy

**Ahmet Okay Caglayan**

Turkey

**Scott Canna**

United States of America

**Ruy Carrasco**

United States of America

**Jeffrey Chaitow**

Australia

**Mercedes Chan**

Canada

**Peter Chira**

United States of America

**Rolando Cimaz**

Italy

**Randy Cron**

United States of America

**Rubén José Cuttica**

Argentina

**Ruben Cuttica**

Spain

**Jaime De Inocencio**

Spain

**Fatma Dedeoglu**

United States of America

**Pavla Dolezalova**

Czech Republic

**Kyla Driest**

United States of America

**Anne Eberhard**

United States of America

**Barbara Eberhard**

United States of America

**Despina Eleftheriou**

United Kingdom

**Raoul Engelbert**

Netherlands

**Fernanda Falcini**

Italy

**Anders Fasth**

Sweden

**Guido Filler**

Canada

**Ivan Foeldvari**

Germany

**Emily Fox**

United States of America

**Caroline Galeotti**

France

**Abraham Gedalia**

United States of America

**Hermann Girschick**

Germany

**Thomas Brent Graham**

United States of America

**Thomas Griffin**

United States of America

**Brandt Groh**

United States of America

**Alexei Grom**

United States of America

**Jaime Guzman**

Canada

**Kathleen Haines**

United States of America

**Najia Hajjaj-Hassouni**

Morocco

**Akiko Hamaoka-Okamoto**

Japan

**Liora Harel**

Israel

**Shunji Hasegawa**

Japan

**Philip Hashkes**

Israel

**Lauren Henderson**

United States of America

**Aimee Hersh**

United States of America

**Gloria Higgins**

United States of America

**Kerry Hitos**

Australia

**Mark Hoeltzel**

United States of America

**Michaël Hofer**

Switzerland

**Matthew Hollander**

United States of America

**Daniel Horton**

United States of America

**Kristin Houghton**

Canada

**Adam Huber**

Canada

**Reagan Hunt**

United States of America

**Maria Ibarra**

United States of America

**Paul Jensen**

United States of America

**Peter Junker**

Denmark

**Leonard Kaban**

United States of America

**Sylvia Kamphuis**

Netherlands

**Susan Kim**

United States of America

**Yukiko Kimura**

United States of America

**Susan Klepper**

United States of America

**Andrea Knight**

United States of America

**Georg Kojda**

Germany

**Rebecca Kunder**

United States of America

**Bianca Lang**

Canada

**Ronald Laxer**

Canada

**Carol Lindsley**

United States of America

**Mindy Lo**

United States of America

**Daniel Lovell**

United States of America

**Karen Marzan**

United States of America

**F Mayouf**

Saudi Arabia

**Deborah McCurdy**

United States of America

**Janet McDonagh**

United Kingdom

**Jay Mehta**

United States of America

**Michael Miller**

United States of America

**Chi Chiu Mok**

Hong Kong

**Terry Moore**

United States of America

**Asha Moudgil**

United States of America

**Kabita Nanda**

United States of America

**Rob Nickeson**

United States of America

**Edward Oberle**

United States of America

**Sheila Oliveira**

Brazil

**Kathleen O'Neil**

United States of America

**Karen Onel**

United States of America

**Murray Passo**

United States of America

**Peri Hickman Pepmueller**

United States of America

**Sampath Prahalad**

United States of America

**Egla Rabinovich**

United States of America

**Athimalaipet Ramanan**

United Kingdom

**Angelo Ravelli**

Italy

**Andreas Reiff**

United States of America

**Sarah Ringold**

United States of America

**Angela Robinson**

United States of America

**Tova Ronis**

United States of America

**Ricardo Russo**

Argentina

**Latasha Ruth**

United States of America

**Marite Rygg**

Norway

**Rotraud Saurenmann**

Switzerland

**Tore Saxne**

Sweden

**Ken Schikler**

United States of America

**Kara Murphy Schmidt**

United States of America

**Yehuda Schoenfeld**

Israel

**L Schwartz**

United States of America

**Susan Shenoi**

United States of America

**Stanford Shulman**

United States of America

**Soumitri Sil**

United States of America

**Anne Stevens**

United States of America

**Matthew Stoll**

United States of America

**Grant Syverson**

United States of America

**Melissa Tesher**

United States of America

**Tracy Ting**

United States of America

**Natasa Toplak**

Slovenia

**Kathryn Torok**

United States of America

**Lori Tucker**

Canada

**Yosef Uziel**

Israel

**Janjaaap van der Net**

Netherlands

**Marielle van Gijn**

Netherlands

**Richard Vehe**

United States of America

**Jelena Vojinovic**

Serbia

**Linda Wagner-Weiner**

United States of America

**Carol Wallace**

United States of America

**Pamela Weiss**

United States of America

**Helmut Wittkowski**

Germany

**Nico Wulffraat**

Netherlands

**Cagri Yildirim Toruner**

United States of America

**Lawrence Zemel**

United States of America

**Dan Zhao**

United States of America

**Vincent Zimmer**

Germany

